# Are Psychological Distress and Resilience Associated with Dietary Intake Among Australian University Students?

**DOI:** 10.3390/ijerph16214099

**Published:** 2019-10-24

**Authors:** Megan C. Whatnall, Amanda J. Patterson, Yu Yao Siew, Frances Kay-Lambkin, Melinda J. Hutchesson

**Affiliations:** 1 School of Health Sciences, Faculty of Health and Medicine, University of Newcastle, Callaghan, NSW 2308, Australia; megan.whatnall@uon.edu.au (M.C.W.); amanda.patterson@newcastle.edu.au (A.J.P.); YuYao.Siew@uon.edu.au (Y.Y.S.); 2 Priority Research Centre for Physical Activity and Nutrition, University of Newcastle, Callaghan, NSW 2308, Australia; 3 Priority Research Centre for Brain and Mental Health, University of Newcastle, Callaghan, NSW 2308, Australia; frances.kaylambkin@newcastle.edu.au

**Keywords:** psychological distress, resilience, dietary intake, diet, university students, college students, mental health

## Abstract

University students report unhealthy diets and experience poorer mental health than the general population. This study explores the association between psychological distress and resilience with dietary intake in a sample of Australian university students. Cross-sectional data from the University of Newcastle Student Healthy Lifestyle Survey 2017 were analysed. Psychological distress (Kessler Scale), resilience (Brief Resilience Scale) and fruit, vegetable, soft drink, takeaway food and breakfast intakes (short diet questions) were assessed. Socio-demographic (e.g., gender), student (e.g., undergraduate/postgraduate) and health characteristics (e.g., physical activity) were captured. Multivariate linear regression models explored associations between psychological distress and resilience with dietary intake, with adjustment for potential confounders. Analysis included 2710 students (mean age 26.9 ± 9.5 years, 30.4% male). In adjusted models, lower psychological distress was associated with higher fruit (β = −0.37, *p* = 0.001) and vegetable (β = −0.37, *p* < 0.001) serves/day, more frequent breakfast consumption (*p* < 0.001) and less frequent soft drink and takeaway food consumption (*p* < 0.001). Higher resilience was associated with higher fruit (β = 0.03, *p* = 0.022) and vegetable (β = 0.06, *p* < 0.001) serves/day, more frequent breakfast consumption (*p* = 0.005), and less frequent soft drink (*p* < 0.001) and takeaway food consumption (*p* = 0.001). These results highlight a potential link between psychological distress and resilience with diet, and that further research in this area is warranted.

## 1. Introduction

University students’ mental health is a growing area of concern, with some evidence indicating a higher prevalence of mental health disorders among university students than in same-aged peers not at university and the general adult population [1,2]. The World Mental Health International College Student project estimated 20–31% of university students are affected by one or more mental health disorders in any given year [3,4]. Rates of other indicators of mental health, such as stress and psychological distress, are also high [2,5]. For instance, among 26,000 students in the Fall 2018 American College Health Assessment survey, 45% reported experiencing more than average stress in the previous year, and 13% reported experiencing tremendous stress [5]. In an Australian study comparing psychological distress among university students and national data from age-matched non-studying peers, the rate of very high psychological distress was 19% among students and 3% among their non-studying peers [1].

The high psychological distress rates among university students may stem from concern around academic factors (study time management and academic performance), as well as environmental factors (managing finances, juggling study with work, family, social and other commitments, changes in living arrangements and managing new social relationships) [2]. The combination of these academic and environmental factors may particularly contribute to the discrepancies in psychological distress observed between studying and non-studying peers [2,6]. For example, Ibrahim et al. suggested university students may experience more stresses in relation to future employment or dissatisfaction with their studies, and that being a student may predispose an individual to depression, due to associated factors such as leaving the family home and a lack of family support [6].

Psychological distress among university students may also be associated with resilience; a measure of the ability to recover from stress [7]. Cross-sectional studies have identified that a low level of resilience pre-dates and predisposes university students to psychological distress [8,9,10,11], consistent with a theoretical model of resilience, coping or regulatory strategies and academic performance posited by Johnson et al. [10]. The model proposes that higher resilience leads to greater use of adaptive regulatory strategies in the face of stress (including time management, effort regulation and self-regulation, including healthy behaviors), which then influences higher academic achievement [10]. On the flipside, low resilience is indirectly associated with lower academic achievement, mediated by a lack of adaptive coping strategies and higher psychological distress. Higher psychological distress among university students has also been associated with unhealthy lifestyle behaviours, including unhealthy diet, excessive consumption of alcohol and smoking [12,13,14].

In the general adult population, observational studies consistently demonstrate that consuming an unhealthy diet is associated with a higher likelihood of mental health problems [15]. Lower intakes of nutrient rich foods, such as fruit and vegetables, and higher intakes of energy-dense nutrient poor foods, are independently associated with higher rates of depression, and higher psychological distress and stress [15,16,17,18,19,20]. Among university students, approximately 45 studies have been conducted to date which explore associations between mental health and diet. These are predominantly cross-sectional analyses from western countries, and have explored associations between diet with depression, anxiety and/or stress. Overall, these studies support that an unhealthy diet is associated with poorer mental health. For example, studies from Canada, France and the UK, found low fruit and vegetable intakes, higher intakes of added sugars, sweets and fast food, as well as irregular meal patterns were associated with higher odds of depression, anxiety and stress [21,22,23].

Most studies exploring diet and mental health are in general adult populations or older adults, and of these, some indicate that the relationship between diet and mental health is moderated by other health behaviours or socio-demographic characteristics, for example physical activity, smoking, body mass index and age [15,24,25]. Further, results from general populations may not be applicable to university students as they are a unique population group, due to elements of their environment (personal and social) that are specifically relevant to the life-stage and the setting [26]. Of the studies among university students, only one has explored the association between diet and psychological distress [27] and none have explored diet and resilience. These are therefore significant gaps in the evidence, and worthy of further investigation given the high rates of both psychological distress and unhealthy diets among university students [1,5,28,29,30], as well as the proposed link between psychological distress and resilience [8,9,10,11]. Further, only two studies have been conducted in Australian university students exploring diet and mental health [31,32]. Papier et al. demonstrated dose-response trends between higher stress levels with higher consumption of unhealthy foods, such as processed foods and alcohol, and lower consumption of healthy foods, such as fruit and vegetables [31]. While Di Benedetto et al., in a cluster analysis of health behaviours and mental health risk, found that higher severity of depression, anxiety, and/or stress and not meeting guidelines for fruit intake clustered together [32]. However, neither of these two studies assessed psychological distress or resilience. Results from studies conducted in different countries, such as Canada, France and the UK may not be applicable to Australia due to differences in culture and dietary recommendations. The aim of this cross-sectional analysis was to explore the associations between psychological distress and resilience with dietary intake in a sample of Australian university students.

## 2. Materials and Methods

### 2.1. Study Design

This study was a secondary analysis of data from an online cross-sectional survey, the 2017 University of Newcastle (UON) Student Healthy Lifestyle Survey (SHLS). The full survey methods and results have been published elsewhere [33]. The aim of the SHLS is to identify lifestyle-related health risk factors, mental health and wellbeing and overweight/obesity prevalence. Study measures were selected to achieve this primary aim. The survey was conducted via Survey Monkey (www.surveymonkey.com.au) and was open from 4 September to 1 October 2017 (during the middle of the semester for most participants). The survey tool allowed access on a single device to prevent multiple entries by the same individual. The survey included 61 questions displayed over 27 pages and took approximately 15 minutes to complete based on pilot testing. Survey questions of a sensitive nature were optional to complete, including drug use, sexual health, and mental health questions. The current analysis specifically focused on mental health and dietary intake data. The study conduct and reporting were compliant with STROBE-nut guidelines [34].

### 2.2. Participants and Recruitment

All students enrolled at the UON as of 4 September 2017 (*n* = 33,783) were invited to participate. Eligibility criteria included current enrolment as a student at the UON. To determine eligibility, a screening question asked individuals if they were a current student. The UON is a large urban university with students based at the main campus in Newcastle, New South Wales (NSW), Australia, additional smaller campuses across NSW (*n* = 4) and online/distance cohorts. Invitations were sent to students via an email to their UON email account on the 4th September 2017, with reminder emails sent on the 13th and 25th of September 2017. University teaching staff were emailed requesting they promote the survey in class or on the online learning management system using the recruitment materials provided by the researchers. The survey was also promoted via UON student social media accounts, digital signage across all campuses and posters at the main campus. On completing the survey, participants could choose to enter a prize draw to win one of five gift vouchers to the value of $AU100. All participants gave informed consent prior to participating. Study approval was obtained from the UON Human Research Ethics Committee (H-2015-0459).

### 2.3. Measures

#### 2.3.1. Dietary Intake

Dietary intake was assessed using short diet questions from the NSW Adult Population Health Survey [35]. This dietary assessment method was selected on this basis of the primary aim of the SHLS from which the current study data was derived for secondary analysis, i.e., an appropriate tool for monitoring and surveillance. The five dietary intake questions analysed in this study were serves per day of fruit (*“Don’t eat”* to *“≥6 serves”)*, where one serve was defined as 150 grams as per the Australian Guide to Healthy Eating (AGHE) [36]; serves per day of vegetables (*“Don’t eat”* to *“≥6 serves”)*, where one serve was defined as 75 grams [36]; consumption frequency of soft drink, cordial or sports drinks (*“≤1 cup/week”* to *“≥2 cups/day”)*, with 1 cup defined as 250 mL; takeaway meals or snacks *(“Never/rarely”* to *“Everyday”*); and breakfast *(“Never/rarely”* to *“Everyday”*). The questions assess usual intake, not a specific reference time period. The fruit and vegetable questions have demonstrated good relative validity and consistency compared with other dietary assessment methods; biomarkers and 24 h recall [37,38]. For fruit and vegetable questions compared with biomarkers, statistically significant associations were found between increasing serum carotenoid and red-cell folate mean concentrations and increasing serves per day categories [38]. The relative validity of the breakfast intake question was deemed poor compared with the 24 h recall method [37], where the validity of the remaining questions has not been assessed.

#### 2.3.2. Psychological Distress

Psychological distress was assessed using the Kessler Psychological Distress Scale (K-10) [39]. The K-10 is a measure of non-specific psychological distress. Participants were asked to rate the frequency with which they experienced each of 10 items, for example, “About how often did you feel hopeless”, over the previous month on a 5-point Likert scale *(1 = None of the time* to 5 = *All of the time)*. The scores for each question sum to a total score between 10–50, which corresponds to the following categories of non-specific psychological distress; low (10–15), moderate (16–21), high (22–29), or very high (30–50) risk.

#### 2.3.3. Resilience

Resilience was assessed using the Brief Resilience Scale (BRS) [7]; a measure of the ability to recover from stress as an underlying psychological trait. Participants were asked to rate their level of agreement with each of six items, for example, “I tend to bounce back quickly after hard times”, on a 5-point Likert scale *(Strongly agree* to *Strongly disagree).* Three of the items are negatively worded and three are positively worded, and responses are scored between 1–5, with a higher score indicating higher resilience. The participants’ average score across the items was calculated and compared with the following categories: low (1.00–2.99), normal (3.00–4.30) or high (4.31–5.00) resilience.

#### 2.3.4. Socio-Demographic and Student Characteristics

Socio-demographic data collected included gender, age, country of birth, Aboriginal or Torres Strait Islander (ATSI) background, marital status, living situation and source(s) of financial support (parents/guardians, partner, government, scholarship, other or none). Student specific data collected included degree type, faculty of study and number of years studying.

#### 2.3.5. Health Behaviours

Physical activity (PA) was assessed using the Active Australia Survey, including the total time (minutes) and number of sessions spent walking and performing moderate and vigorous activity in the previous week [40]. PA data were compared with national recommendations (≥150 minutes moderate activity/week over ≥five sessions) to determine sufficiency [41]. Sitting time was assessed using questions from the NSW Adult Population Health Survey, including average sitting time on a weekend day and on a weekday [35]. The average total sitting time was then calculated as [(total time spent sitting on a weekday × 5) + (total time spent sitting on a weekend day × 2)/7]. Smoking status was assessed and categorised into smokers (those indicating they smoke daily or occasionally) and non-smokers (all other responses) [35]. Alcohol and drug use were assessed via the Alcohol Use Disorders Identification Test (AUDIT) and Drug Use Disorders Identification Test (DUDIT), respectively [42,43]. AUDIT and DUDIT are screening tools to identify and grade alcohol and drug abuse or risk of. The cut points for the AUDIT include abstinence/low risk (0–7), moderate risk (8–15), harmful/hazardous use (16–19), or dependence (20–40). The cut points for the DUDIT are sex specific and include no drug related problems (males 0–5, females 0–1), drug related problems (males 6–24, females 2–24), or heavily dependent on drugs (25–44). Sleep was assessed via a question from the National Centre for Chronic Disease Prevention and Health Promotion, where participants reported their average hours of sleep in a 24-hr period [44]. Average hours of sleep were then compared with the Sleep Health Foundation age-based recommendations [44], these include 8–10 hours for 17-year olds, 7–9 hours for 18–64 year olds, and 7–8 hours for those ≥65 years. Body mass index (BMI) was calculated using the standard equation (weight (kg)/height (m^2^)) from participants reported height in cm and weight in kg, and compared with the World Health Organization BMI cut points [45].

#### 2.3.6. Statistical Analysis

Analysis was conducted using STATA statistical software version 14.1. Demographic and health characteristics, dietary intake, psychological distress and resilience were described as percentages for categorical variables and means and standard deviations (SD) for continuous variables. A total of 3529 individuals responded to the survey, of which 3464 consented and were eligible to participate, and 3076 completed all compulsory questions. The total included in this analysis was 2710. Participants were excluded from the analysis if they were missing data on psychological distress (*n* = 114), resilience (*n* = 30), drug use (*n* = 35) or BMI (*n* = 26), or due to implausible data on physical activity (*n* = 160) or sleep (*n* = 1). Differences in demographic characteristics between those included in this analysis and those who chose not to complete the psychological distress and resilience questions were also assessed, with no significant differences found. Unadjusted linear regression models were used to explore associations between psychological distress (Kessler total score) and resilience (Brief Resilience Scale total score) with sociodemographic and student characteristics, health behaviours and dietary intake (vegetable, fruit, soft drink, takeaway foods and breakfast), to identify potential confounders. Statistical significance for the identification of potential confounders was set at *p* < 0.2. Fully adjusted linear regression models were used to explore associations between psychological distress and resilience with dietary intake, including the demographic and health characteristics found to be significant in the unadjusted models as potential confounders. Due to the large number of potential confounders identified and to prevent over-adjustment, backwards stepwise regression with comparison of Bayesian information criterion was used to eliminate potential confounders not contributing to the models and identify the models with best fit. Statistical significance for the fully adjusted models was set at *p* < 0.05. For measures with both a continuous and a categorical variable (age, physical activity, sleep, alcohol use, drug use and BMI), the continuous variable was included in fully adjusted models.

## 3. Results

### 3.1. Summary of Sample Characteristics

Participants’ mean ± SD age was 26.9 ± 9.5 years (Table 1). The majority (56.4%) were aged 17–24 years, which is consistent with university students across Australia (64% aged 17–24 years) [46]. Most participants were female (69.1%) and born in Australia (82.0%). Participants were undergraduate (70.7%), postgraduate (21.8%) and enabling (i.e., transition to university) and English language course students (7.5%). Most students were living in private rental accommodation off-campus (40.6%) or their parents’ home (33.2%). Students were from all five faculties across the university, with the largest proportions from Health and Medicine (31.0%) and Education and Arts (24.5%) faculties. Approximately half of the participants (51.0%) consumed 2 or more serves per day of fruit, while only 11.6% consumed 5 or more serves per day of vegetables. The majority of the participants consumed 1 cup or less per week of soft drink, cordial or sports drinks (74.0%) and consumed takeaway foods less than one day per week (69.3%), while just over half (58.2%) consumed breakfast every day.

### 3.2. Summary of Psychological Distress and Resilience

The percentage of participants in each category of non-specific psychological distress risk was 30.8% low, 32.0% moderate, 22.9% high and 14.3% very high risk. The percentage of participants with low resilience was 29.7%, with 59.9% classified as normal resilience, and 10.4% high resilience. The mean ± SD psychological distress risk score was 20.6 ± 7.8 out of 50, and the mean ± SD resilience score was 3.3 ± 0.8 out of 5. Unadjusted linear regression of psychological distress and resilience found that lower psychological distress score was significantly associated with higher resilience score (β = −5.716, *p* < 0.001).

### 3.3. Associations between Psychological Distress and Resilience with Dietary Intake

Results of the unadjusted models for psychological distress and resilience with dietary intake are presented in Table 2. Results of the unadjusted models for psychological distress and resilience with demographic and health characteristics (potential confounders) are presented in Table 1. Fully adjusted models for psychological distress and resilience with dietary intake are presented in Table 3, with the adjustments for potential confounders described in the footnote. Supplementary Table S1 includes the full results of the adjusted models for psychological distress and resilience with dietary intake, i.e., including the potential confounders adjusted for.

In the adjusted models (Table 3), controlling for demographic and health characteristics, lower psychological distress score (i.e., lower risk of non-specific psychological distress) was significantly associated with higher serves per day of vegetables (β = −0.368, *p* < 0.001) and fruit (β = −0.374, *p* = 0.001), higher frequency of breakfast consumption (*p* < 0.001), and lower frequencies of soft drink, cordial or sports drink (*p* < 0.001) and takeaway food (*p* < 0.001) consumption.

In the adjusted models, higher resilience score (i.e., higher resilience) was significantly associated with higher serves per day of vegetables (β = 0.055, *p* < 0.001) and fruit (β = 0.028, *p* = 0.022), higher frequency of breakfast consumption (*p* = 0.005), and lower frequencies of soft drink, cordial or sports drink (*p* < 0.001) and takeaway food (*p* = 0.001) consumption.

## 4. Discussion

This cross-sectional study investigated the associations between psychological distress, resilience and dietary intake in a sample of Australian university students. Thirty-seven percent of the sample had high or very risk of psychological distress, and 30% had low resilience. Fifty percent of the sample consumed 2 or more serves per day of fruit, and 11% consumed 5 or more serves per day of vegetables. Around 70% of the sample each consumed 1 cup or less per week of soft drink and consumed takeaway foods less than one day per week. Fifty eight percent consumed breakfast every day. Associations were found between lower risk of psychological distress and higher resilience with higher intakes of vegetables and fruit, more frequent breakfast consumption, and less frequent intakes of soft drink and takeaway food.

The present study found that psychological distress scores, on a score range of 10–50, were 0.37 points lower for each additional serve/day of vegetables or fruit, and 3.00 points lower for consumption of breakfast everyday compared with less than once/week. It was also found that psychological distress scores were 2.39 points higher for consumption of two or more cups/day of soft drink compared with one cup or less/week, and 9.49 points higher for consumption of takeaway food everyday compared with less than once/week. These findings are consistent with the study by Knowlden et al. of 195 university students from the USA, which used the similar 6-item Kessler Scale to assess psychological distress [27]. Knowlden et al. found that participants with low compared with severe psychological distress risk consumed significantly higher quantities of fruit and significantly lower quantities of sugar-sweetened beverages, however, found no significant differences in psychological distress risk related to vegetable intake. There is also consistency between the current study findings with studies among university students and young adults for other mental health outcomes. That is, other studies have found associations between higher intakes of fruit and vegetables with lower risk of depression and stress [21,23,31], and between higher intakes of added sugars, sweets, fast food and processed foods with higher risk of depression, stress and anxiety, and lower wellbeing [21,23,31,47].

The present study also found that resilience scores, on a range of 1–5, were 0.06 and 0.03 points higher for each additional serve/day of vegetables and fruit respectively, and 0.14 points higher for consumption of breakfast everyday compared with less than once/week. It was also found that resilience scores were 0.24 points lower for consumption of two or more cups/day of soft drink compared with one cup or less/week, and 0.38 points lower for consumption of takeaway food 5–6 times/week compared with less than once/week. As this is one of the first studies to assess resilience in relation to diet, studies to compare with are limited. However, these findings are somewhat consistent with a study of 1853 Chinese university students by Chu et al., where students who placed a greater importance on nutrition had a higher sense of coherence (a measure of an individual’s sense of optimism and control) [48]. Additionally, the associations between dietary behaviours and resilience observed in the current study were the inverse of that between dietary behaviours and psychological distress, which is consistent with previous studies reporting an inverse relationship between psychological distress and resilience [2,10].

There is a growing body of evidence to support the link between a healthy diet and better mental health outcomes [15,49]. Much of the evidence comes from cross-sectional studies, and some studies have found no association when controlling for potential confounding factors [15]. However, there is now more evidence emerging from cohort and intervention studies, and the overall evidence supports that a link exists [50,51]. In terms of the direction of the relationship between diet and mental health, this is a complex and likely bi-directional relationship [52]. There are indications for a healthy diet contributing to improved mental health. For example, Sarris et al. suggests that it is the reliance of the brain on the necessary nutrients to function, including immune, antioxidant defence and neurotrophic systems, that implicate diet in the pathogenesis of mental health disorders [53]. Conversely, there are indications that dietary choices are influenced by mental health status. For example, studies have shown that individuals often make less healthy dietary choices during periods of stress or to alleviate feelings of stress [52]. The relationship between diet and mental health is complex with many more factors at play and requires further investigation among a range of population groups, including university students.

The National Youth Health Foundation mental health service Headspace, is a mental health service aimed at early intervention for mental health related issues, with the most common intervention being cognitive behavioural therapy (CBT), and an average of four intervention sessions per individual [54,55]. Analyses of data from Australian youth (aged 12–25 years) attending Headspace calculated a Reliable Change Index (RCI) as a 7-point change in K-10 score from first to last intervention session [54,55]. The magnitude of the associations between dietary intake and K-10 scores in the present study are relatively small in comparison to the RCI, although they vary across the dietary intake measures. The findings suggest that there is a small, yet positive link between diet and psychological distress, and improving one might have a positive effect on the other.

Another interesting point of comparison is the magnitude of the association between mental health outcomes across the different dietary intake outcomes assessed. In the current study, larger estimates were found for the associations of soft drink, takeaway and breakfast with psychological distress and resilience than for fruit and vegetables. Similarly, Knowlden et al. reported a larger association between sugar-sweetened beverages and psychological distress than for fruit [27]. In contrast, El Ansari et al. found greater associations for fruit and vegetables than for sweets and fast food in relation to stress, while for depression, the findings differed for males and females [21]. As for why certain foods or dietary components may have larger association than others, this could be related to the strength of the underlying mechanisms behind the associations between diet and mental health. These are proposed to be similar to the mechanisms between diet and cognition and cardiovascular diseases [56]. Nutrients that are known to have beneficial effects on mental health include those which protect against oxidative stress, such as Vitamins B6, B9 and B12, due to their interactions with homocysteine, and the antioxidant vitamins A, C and E [56], as well as polyphenols and flavonoids [57,58]. Fruits and vegetables are rich sources of these, which could explain the findings of lower psychological distress and higher resilience with higher fruit and vegetable intake. Nutrients with negative effects on mental health are those with pro-inflammatory effects, including saturated fats and sugar when consumed in excess [56,59,60]. The findings of higher psychological distress and lower resilience with higher intakes of takeaway and soft drink are in line with this.

As with all cross-sectional surveys, the direction of the association between psychological distress, resilience and dietary intake cannot be determined from these data, nor causality. In addition, the use of short diet questions to assess dietary intake is limiting in terms of validity compared with other dietary assessment methods, such as validated food frequency questionnaire. However, this dietary assessment method was selected on the basis of the primary aim of the survey, i.e., monitoring and surveillance, and is still an appropriate method for this study. Further, a small number of dietary behaviours were assessed. It is also a limitation that body dissatisfaction and/or eating-disordered behaviour were not assessed in this study as associations have been found between these factors and dietary intake and mental health [61,62,63]. Therefore, if body dissatisfaction and eating-disordered behaviour were accounted for as potential confounding factors this may have attenuated the current findings. A further limitation is the measures being self-reported. However, the use of an online, anonymous platform for collecting survey data and validated tools minimize the potential bias from self-reporting. The survey was conducted at a time during semester (i.e., the middle) that is likely to be representative of most student’s typical psychological distress, resilience and dietary intake during the university year. Periods of higher academic pressure, such as exam periods were avoided. Further, the sample was a small proportion of the total UON student body (8.0%). In terms of representativeness, the sample consisted of slightly higher proportions of female, undergraduate and domestic students compared with the average across Australian universities, however sample characteristics were otherwise consistent [46]. The representation of ATSI students was higher, however proportional to UON numbers [64]. The representativeness of the sample in terms of dietary intake, psychological distress and resilience is not able to be determined as this data does not exist for all UON students or all Australian university students. Compared with other studies among university students and with data from Australian young adults, this study sample were consuming soft drinks and takeaway foods less frequently, and a greater proportion were meeting guidelines for fruit and vegetable intake [32,65,66,67]. The proportion with high or very high psychological distress in the current study was similar to an earlier study among Australian university students [1]. As such, the study sample appears to be healthier than university students more generally in terms of dietary intake, and this should be considered in terms of representativeness and generalisability. Despite these limitations, the present analysis is important as it suggests that an association exists in this population group, and that further enquiry of this association is warranted. The main strengths of the present study include the use of validated tools to measure psychological distress and resilience [7,39], the large number of potential confounders accounted for in analyses, and the large sample size.

## 5. Conclusions

This cross-sectional analysis highlights a potential link between psychological distress and resilience with diet, however further research is needed to substantiate the findings. Future studies should include cohort studies to track changes in mental health and diet and their interrelationships prospectively. Further studies should also conduct more comprehensive evaluation of diet and using validated dietary assessment tools. In addition, it would be worthwhile to study the associations between diet and mental health in university students compared with matched non-studying peers. Longer term, the data collected from this and future studies of psychological distress, resilience and diet could be useful to inform mental health and/or dietary interventions to support university students.

## Figures and Tables

**Table 1:** Mean psychological distress and resilience scores of a sample of Australian university students by demographic and health characteristics (*n* = 2710).

Variable	*N* or Mean	% or SD	K10 Score ^a^ (Mean ± SD)	BRS Score ^b^ (Mean ± SD)
Gender *^,†^				
Male	824	30.4	19.5 ± 7.5	3.5 ± 0.8
Female	1872	69.1	21.0 ± 7.9	3.2 ± 0.8
Another gender identity	14	0.5	25.2 ± 7.7	3.0 ± 0.7
Age (years) (Mean ± SD) *^,†^	26.9	9.5		
≤20 years old	688	25.4	21.9 ± 8.1	3.2 ± 0.8
21–24 years	841	31.0	21.4 ± 7.7	3.2 ± 0.8
25–29 years	475	17.5	21.2 ± 8.2	3.3 ± 0.8
30–39 years	398	14.7	18.7 ± 7.0	3.4 ± 0.8
≥40 years	308	11.4	16.9 ± 6.2	3.6 ± 0.8
Country of birth ^c,^*^,†^				
Australia	2215	82.0	21.0 ± 7.9	3.3 ± 0.8
Other	486	18.0	18.6 ± 7.3	3.4 ± 0.8
Aboriginal or Torres Strait Islander background *^,†^				
Yes	75	2.8	24.1 ± 9.6	3.0 ± 1.0
No	2635	97.2	20.5 ± 7.7	3.3 ± 0.8
Marital Status *^,†^				
Never married	1858	68.6	21.4 ± 8.0	3.2 ± 0.8
Married	458	16.9	17.3 ± 6.0	3.5 ± 0.8
Defacto	287	10.6	20.6 ± 7.6	3.0 ± 0.9
Separated	35	1.3	21.2 ± 9.8	3.5 ± 0.9
Divorced	67	2.5	20.2 ± 8.9	3.5 ± 0.8
Widowed	5	0.2	13.6 ± 3.4	4.3 ± 0.6
Living Situation *^,†^				
Own home	410	15.1	17.4 ± 6.1	3.6 ± 0.8
Parents home	900	33.2	21.6 ± 7.7	3.2 ± 0.8
On-campus	223	8.2	20.8 ± 7.7	3.3 ± 0.8
Renting	1099	40.6	20.7 ± 8.0	3.3 ± 0.8
Boarding/Homestay	53	2.0	23.5 ± 9.3	3.1 ± 0.9
Irregular	25	0.9	25.1 ± 9.9	2.9 ± 0.9
Receiving financial support *^,†^				
Yes	1700	62.7	21.1 ± 7.9	3.2 ± 0.8
No	1010	37.3	19.7 ± 7.6	3.4 ± 0.8
Type of degree *^,†^				
Undergraduate	1917	70.7	21.2 ± 7.9	3.3 ± 0.8
Postgraduate	591	21.8	17.9 ± 6.5	3.5 ± 0.8
Other ^d^	202	7.5	22.2 ± 9.0	3.2 ± 0.8
Faculty of Study *^,†^				
Business and Law	363	13.4	20.1 ± 7.7	3.4 ± 0.8
Education and Arts	663	24.5	21.8 ± 8.4	3.2 ± 0.8
Engineering	327	12.1	20.3 ± 7.4	3.4 ± 0.8
Health and Medicine	839	31.0	19.3 ± 7.1	3.4 ± 0.8
Science	370	13.7	21.2 ± 7.8	3.3 ± 0.8
English Language and Foundation Studies	148	5.5	22.8 ± 9.1	3.1 ± 0.8
**Number of years studying**				
1st year	1055	38.9	21.0 ± 8.0	3.3 ± 0.8
2nd year	553	20.4	20.5 ± 8.0	3.3 ± 0.9
3rd year	530	19.6	20.4 ± 7.6	3.3 ± 0.8
4th year	299	11.0	20.3 ± 7.3	3.4 ± 0.8
5th year or later	273	10.1	20.1 ± 7.7	3.3 ± 0.8
Physical activity (mins/week) (Mean ± SD) *^,†^	350	307		
Meeting physical activity recommendations *^,†^				
Yes	1685	62.2	20.0 ± 7.7	3.2 ± 0.8
No	1025	37.8	21.5 ± 8.0	3.4 ± 0.8
Smoking Status *^,†^				
Smoker	182	6.7	23.9 ± 8.9	3.1 ± 0.9
Non-Smoker	2528	93.3	20.3 ± 7.7	3.3 ± 0.8
Sleep (hours/24h period) (Mean ± SD) *	7	1		
Meeting sleep recommendations *^,†^				
No	691	25.5	22.8 ± 8.8	3.2 ± 0.9
Yes	2019	74.5	19.8 ± 7.3	3.3 ± 0.8
Average sitting time (mins/day) *^,†^ (Mean ± SD)	494	283		
AUDIT score (Mean ± SD) *^,†^	5.2	5.2		
AUDIT classification *^,†^				
Abstinence/low risk	2027	74.8	20.0 ± 7.7	3.3 ± 0.8
Moderate risk	549	20.3	21.9 ± 7.8	3.3 ± 0.8
Harmful/hazardous use	75	2.8	23.6 ± 8.5	3.1 ± 0.8
Dependence	59	2.2	26.0 ± 8.9	3.0 ± 0.9
DUDIT score (Mean ± SD) *,†	1.1	3.3		
DUDIT classification ^e,^*^,†^				
No drug related problems	2301	85.4	20.1 ± 7.7	3.3 ± 0.8
Drug related problems	384	14.2	23.0 ± 7.7	3.1 ± 0.8
Heavily dependent on drugs	11	0.4	35.9 ± 8.7	2.2 ± 1.0
BMI (kg/m^2^) (Mean ± SD) *^,†^	25.2	5.9		
BMI cut points *^,†^				
<18 kg/m^2^: Underweight	131	4.8	21.4 ± 8.5	3.2 ± 0.9
18–24.9 kg/m^2^: Healthy weight	1517	56.0	19.9 ± 7.4	3.3 ± 0.8
3.325–29.9 kg/m^2^: Overweight	640	23.6	20.6 ± 7.9	3.3 ± 0.8
>30 kg/m^2^: Obese	422	15.6	22.7 ± 8.7	3.2 ± 0.9

^a^ K-10 score range 10–50, higher score indicates higher psychological distress. ^b^ BRS score range 1–5, higher score indicates higher resilience. ^c^ *n* = 2701 (*n* = 9 unspecified). ^d^ Includes students enrolled in enabling (i.e., transition to university) courses and English language courses for international students. ^e^ *n* = 2696 as classifications are sex specific (*n* = 14 participants indicated other gender identity). * Indicates statistically significant difference in psychological distress at *p* < 0.2 level. ^†^ Indicates statistically significant difference in resilience at *p* < 0.2 level.

**Table 2:** Mean psychological distress and resilience scores of a sample of Australian university students by dietary intake (*n* = 2710).

Variable	*N*	%	K10 Score ^a^ (Mean ± SD)	BRS Score ^b^ (Mean ± SD)
Vegetable (serves/day) *^,†^				
0	12	0.4	23.8 ± 6.4	3.2 ± 0.8
<1	195	7.2	22.5 ± 8.7	3.1 ± 0.9
1	437	16.1	21.4 ± 8.5	3.2 ± 0.8
2	738	27.2	20.8 ± 7.7	3.3 ± 0.8
3	644	23.8	20.3 ± 7.4	3.3 ± 0.8
4	369	13.6	20.0 ± 7.4	3.4 ± 0.8
5	184	6.8	19.3 ± 7.3	3.4 ± 0.8
6 or more	131	4.8	18.4 ± 7.6	3.6 ± 0.9
Fruit (serves/day) *^,†^				
0	70	2.6	23.4 ± 8.8	3.3 ± 1.0
<1	447	16.5	22.0 ± 8.6	3.2 ± 0.9
1	811	29.9	20.8 ± 7.7	3.3 ± 0.8
2	828	30.6	19.9 ± 7.4	3.3 ± 0.8
3	378	14.0	19.9 ± 7.7	3.4 ± 0.8
4	112	4.1	19.8 ± 7.0	3.4 ± 0.8
5	33	1.2	19.2 ± 8.4	3.4 ± 0.9
6 or more	31	1.1	20.4 ± 9.1	3.3 ± 1.0
Soft drink *^,†^				
1 cup or less per week	2006	74.0	19.9 ± 7.4	3.3 ± 0.8
2–6 cups per week	514	19.0	22.0 ± 8.2	3.2 ± 0.8
1 cup per day	99	3.7	23.7 ± 9.1	3.0 ± 0.8
2 or more cups per day	91	3.4	24.1 ± 9.8	3.0 ± 0.8
Takeaway food *^,†^				
Less than once per week	1879	69.3	19.7 ± 7.5	3.4 ± 0.8
1–2 times per week	673	24.8	22.2 ± 8.2	3.2 ± 0.8
3–4 times per week	132	4.9	23.2 ± 7.9	3.1 ± 0.8
5–6 times per week	23	0.9	25.9 ± 10.4	2.9 ± 0.8
Everyday	3	0.1	34.7 ± 9.5	3.1 ± 1.0
Breakfast *^,†^				
Less than once per week	268	9.9	23.9 ± 9.3	3.1 ± 0.8
1–2 times per week	234	8.6	22.0 ± 8.2	3.3 ± 0.8
3–4 times per week	299	11.0	21.5 ± 8.3	3.2 ± 0.8
5–6 times per week	332	12.3	21.6 ± 7.4	3.2 ± 0.8
Everyday	1577	58.2	19.4 ± 7.2	3.4 ± 0.8

^a^ K-10 score range 10–50, higher score indicates higher psychological distress. ^b^ BRS score range 1–5, higher score indicates higher resilience. * Indicates statistically significant difference in psychological distress at *p* < 0.2 level. ^†^ Indicates statistically significant difference in resilience at *p* < 0.2 level.

**Table 3:** Linear regression results of psychological distress and resilience scores with dietary intake in a sample of Australian university students (*n* = 2710).

	Psychological Distress Score			Resilience Score		
	β-Coefficient ^a^	SE	*p*	β-Coefficient ^a^	SE	*p*
**Vegetable (serves/day)**	−0.368	0.096	**<0.001 ^b^**	0.055	0.010	**<0.001 ^d^**
**Fruit (serves/day)**	−0.374	0.113	**0.001 ^b^**	0.028	0.012	**0.022 ^e^**
**Soft drink**			**<0.001 ^c^**			**<0.001 ^f^**
Reference category = 1 cup or less per week						
2–6 cups per week	1.409	0.360	<0.001	−0.095	0.039	0.014
1 cup per day	2.471	0.742	0.001	−0.223	0.080	0.005
2 or more cups per day	2.387	0.785	0.002	−0.235	0.084	0.005
**Takeaway food**			**<0.001 ^b^**			**0.001 ^e^**
Reference category = Less than once per week						
1–2 times per week	1.441	0.332	<0.001	−0.124	0.035	<0.001
3–4 times per week	1.279	0.661	0.053	−0.129	0.071	0.069
5–6 times per week	3.613	1.514	0.017	−0.380	0.162	0.019
Everyday	9.494	4.162	0.023	0.031	0.446	0.944
**Breakfast**			**<0.001**			**0.005 ^e^**
Reference category = Less than once per week						
1–2 times per week	−1.228	0.637	0.054	0.137	0.069	0.046
3–4 times per week	−2.078	0.599	0.001	0.076	0.064	0.237
5–6 times per week	−1.780	0.588	0.002	0.002	0.063	0.978
Everyday	−3.004	0.481	<0.001	0.142	0.051	0.006

^a^ β-Coefficient indicates the increase in psychological distress or resilience score per unit increase in the dietary intake variable. Higher psychological distress score indicates higher psychological distress, higher resilience score indicates higher resilience. ^b^ Models adjusted for age, gender, Aboriginal or Torres Strait Islander background, living situation, financial support, type of degree, faculty of study, physical activity time, smoking, sleep time, sitting time, AUDIT score, DUDIT score, and BMI. ^c^ Models adjusted for age, gender, Aboriginal or Torres Strait Islander background, living situation, financial support, type of degree, faculty of study, physical activity time, sleep time, sitting time, AUDIT score, DUDIT score, and BMI. ^d^ Models adjusted for age, gender, marital status, living situation, financial support, faculty of study, physical activity time, DUDIT score, and BMI. ^e^ Models adjusted for age, gender, living situation, financial support, faculty of study, physical activity time, DUDIT score, and BMI. ^f^ Models adjusted for age, gender, living situation, financial support, faculty of study, physical activity time, AUDIT score, DUDIT score, and BMI. Significant *p*-values in **bold**.

## Supplementary Materials

The following are available online at https://www.mdpi.com/1660-4601/16/21/4099/s1, Table S1: Linear regression results of psychological distress and resilience scores with dietary intake in a sample of Australian university students (*n* = 2710), including full results of confounder variables.
