# Supplementary material for: Are Psychological Distress and Resilience Associated with Dietary Intake Among Australian University Students?
Source: Int J Environ Res Public Health. 2019 Oct 24;16(21):4099. doi: 10.3390/ijerph16214099 (PMC6862530; doi:10.3390/ijerph16214099)
Supplement: Supplementary file 1 [file ijerph-16-04099-s001.pdf]

Supplementary Table S1 Linear regression results of psychological distress and resilience scores with dietary intake in a sample of Australian university students (n = 2710), including full results of confounder variables

	Psychological distress score			Resilience score		
	**β-Coefficient[a]**	**SE**	***p***	**β-Coefficient[a]**	**SE**	***p***
Vegetable (serves/day)	-0.368	0.096	**<0.001**	0.055	0.010	**<0.001**
**Gender**			**<0.001**			**<0.001**
Reference category = female						
Male	-2.427	0.328	<0.001	0.329	0.035	<0.001
Another gender identity	2.531	1.920	0.188	-0.002	0.205	0.992
**Age**	-0.125	0.020	**<0.001**	0.008	0.002	**0.001**
**Non-ATSI**	-2.390	0.843	**0.005**			
**Marital status**						**0.022**
Reference category = Never married						
Married				0.024	0.053	0.643
Defacto				-0.112	0.052	0.031
Separated				0.069	0.134	0.608
Divorced				0.090	0.105	0.389
Widowed				0.840	0.344	0.015
**Living situation**			**<0.001**			**<0.001**
Reference category = Parents home						
Own home	-1.655	0.537	0.002	0.265	0.062	<0.001
On-campus	-1.237	0.551	0.025	0.083	0.058	0.153
Renting	-0.167	0.347	0.630	0.082	0.038	0.031
Boarding/Homestay	1.519	1.013	0.134	-0.034	0.108	0.754
Irregular	3.027	1.457	**0.038**	-0.211	0.156	0.175
**Not receiving financial support**	-0.800	0.297	**0.007**	0.089	0.032	**0.005**
**Type of degree**			**0.002**			
Reference category = Undergraduate						
Postgraduate	-1.318	0.378	<0.001			
Other [b]	-0.730	0.901	0.418			
**Faculty of study**			**<0.001**			**<0.001**
Reference category = Health and Medicine						
Business and Law	0.488	0.456	0.285	-0.062	0.048	0.201
Education and Arts	1.850	0.375	<0.001	-0.198	0.040	<0.001
Engineering	1.266	0.498	0.011	-0.155	0.053	0.003
Science	1.273	0.452	0.005	-0.134	0.048	0.006
English Language and Foundation Studies	2.343	1.057	0.027	-0.203	0.069	0.003
**Physical activity (mins/week)**	-0.001	0.0004	**0.006**	0.0002	0.0001	**<0.001**
**Non-smoker**	-1.201	0.588	**0.041**			
**Sleep (hours/24hr period)**	-0.636	0.106	**<0.001**			
**Average sitting time (mins/day)**	0.002	0.001	**0.001**			
**AUDIT score**	0.160	0.029	**<0.001**			
**DUDIT score**	0.289	0.047	**<0.001**	-0.028	0.005	**<0.001**
**BMI (kg/m²)**	0.158	0.024	**<0.001**			
Fruit (serves/day)	-0.374	0.113	**0.001**	0.028	0.012	**0.022**
**Gender**			**<0.001**			**<0.001**
Reference category = female						
Male	-2.310	0.325	<0.001	0.302	0.035	<0.001
Another gender identity	2.546	1.922	0.185	0.006	0.206	0.976
**Age**	-0.127	0.020	**<0.001**	0.010	0.002	**<0.001**
**Non-ATSI**	-2.465	0.844	**0.004**			
**Living situation**			**<0.001**			**<0.001**
Reference category = Parents home						
Own home	-1.738	0.537	0.001	0.264	0.057	<0.001
On-campus	-1.114	0.552	0.044	0.075	0.058	0.200
Renting	-0.175	0.347	0.614	0.067	0.037	0.067
Boarding/Homestay	1.614	1.014	0.112	-0.060	0.109	0.579
Irregular	2.844	1.458	0.051	-0.199	0.156	0.203

**Not receiving financial support**	-0.783	0.298	**0.009**	0.087	0.032	**0.006**
**Type of degree**			**0.003**			
Reference category = Undergraduate						
Postgraduate	-1.290	0.378	0.001			
Other [b]	-0.551	0.902	0.541			
**Faculty of study**			**<0.001**			**<0.001**
Reference category = Health and Medicine						
Business and Law	0.584	0.456	0.200	-0.074	0.049	0.125
Education and Arts	1.959	0.375	<0.001	-0.216	0.040	<0.001
Engineering	1.316	0.498	0.008	-0.158	0.053	0.003
Science	1.266	0.453	0.005	-0.141	0.048	0.004
English Language and Foundation Studies	2.414	1.058	0.023	-0.235	0.069	0.001
**Physical activity (mins/week)**	-0.001	0.001	**0.004**	0.0003	0.0001	**<0.001**
**Non-smoker**	-1.240	0.588	**0.035**			
**Sleep (hours/24hr period)**	-0.637	0.106	**<0.001**			
**Average sitting time (mins/day)**	0.002	0.0004	**<0.001**			
**AUDIT score**	0.159	0.030	**<0.001**			
**DUDIT score**	0.285	0.047	**<0.001**	-0.028	0.005	**<0.001**
**BMI (kg/m²)**	0.157	0.025	**<0.001**	-0.012	0.003	**<0.001**
Soft drink			**<0.001**[c]			**<0.001**
Reference category = 1 cup or less per week						
2-6 cups per week	1.409	0.360	<0.001	-0.095	0.039	0.014
1 cup per day	2.471	0.742	0.001	-0.223	0.080	0.005
2 or more cups per day	2.387	0.785	0.002	-0.235	0.084	0.005
**Gender**			**<0.001**			**<0.001**
Reference category = female						
Male	-2.515	0.328	<0.001	0.326	0.035	<0.001
Another gender identity	2.256	1.918	0.240	0.021	0.206	0.921
**Age**	-0.118	0.020	**<0.001**	0.008	0.002	**<0.001**
**Non-ATSI**	-2.318	0.842	**0.006**			
**Living situation**			**<0.001**			**<0.001**
Reference category = Parents home						
Own home	-1.670	0.536	0.002	0.260	0.057	<0.001
On-campus	-1.117	0.551	0.043	0.092	0.059	0.118
Renting	-0.066	0.347	0.849	0.062	0.037	0.092
Boarding/Homestay	1.573	1.012	0.120	-0.055	0.108	0.610
Irregular	2.972	1.455	0.041	-0.201	0.156	0.198
**Not receiving financial support**	-0.820	0.297	**0.006**	0.095	0.032	**0.003**
**Type of degree**			**0.007**			
Reference category = Undergraduate						
Postgraduate	-1.194	0.378	0.002			
Other [b]	-0.439	0.899	0.625			
**Faculty of study**			**<0.001**			**<0.001**
Reference category = Health and Medicine						
Business and Law	0.522	0.455	0.251	-0.066	0.049	0.173
Education and Arts	1.879	0.374	<0.001	-0.208	0.040	**<0.001**
Engineering	1.312	0.497	0.008	-0.160	0.053	0.002
Science	1.283	0.452	0.005	-0.142	0.048	0.003
English Language and Foundation Studies	1.948	1.059	0.066	-0.197	0.069	0.005
**Physical activity (mins/week)**	-0.002	0.001	**0.001**	0.0003	0.0001	**<0.001**
**Sleep (hours/24hr period)**	-0.610	0.106	**<0.001**			
**Average sitting time (mins/day)**	0.002	0.001	**<0.001**			
**AUDIT score**	0.172	0.029	**<0.001**	-0.007	0.003	**0.032**
**DUDIT score**	0.302	0.045	**<0.001**	-0.023	0.005	**<0.001**
**BMI (kg/m²)**	0.153	0.025	**<0.001**	-0.011	0.003	**<0.001**
Takeaway food			**<0.001**			**0.001**
Reference category = Less than once per week						
1-2 times per week	1.441	0.332	<0.001	-0.124	0.035	<0.001
3-4 times per week	1.279	0.661	0.053	-0.129	0.071	0.069

5-6 times per week	3.613	1.514	0.017	-0.380	0.162	0.019
Everyday	9.494	4.162	0.023	0.031	0.446	0.944
**Gender**			**<0.001**			**<0.001**
Reference category = female						
Male	-2.423	0.326	**<0.001**	0.315	0.035	<0.001
Another gender identity	2.497	1.919	0.193	0.016	0.206	0.939
**Age**	-0.119	0.020	**<0.001**	0.009	0.002	<0.001
**Non-ATSI**	-2.182	0.844	0.010			
**Living situation**			**<0.001**			<0.001
Reference category = Parents home						
Own home	-1.679	0.536	0.002	0.259	0.057	<0.001
On-campus	-1.234	0.551	0.025	0.084	0.058	0.148
Renting	-0.171	0.347	0.622	0.065	0.037	0.078
Boarding/Homestay	1.416	1.011	0.161	-0.043	0.109	0.693
Irregular	2.890	1.454	0.047	-0.201	0.156	0.197
**Not receiving financial support**	-0.835	0.297	0.005	0.090	0.032	0.005
**Type of degree**			**0.004**			
Reference category = Undergraduate						
Postgraduate	-1.262	0.377	0.001			
Other [b]	-0.510	0.901	0.571			
**Faculty of study**			**<0.001**			**<0.001**
Reference category = Health and Medicine						
Business and Law	0.474	0.455	0.298	-0.065	0.049	0.184
Education and Arts	1.924	0.374	<0.001	-0.213	0.040	<0.001
Engineering	1.387	0.498	0.005	-0.168	0.053	0.002
Science	1.313	0.452	0.004	-0.145	0.048	0.003
English Language and Foundation Studies	1.999	1.062	0.060	-0.213	0.069	0.002
**Physical activity (mins/week)**	-0.001	0.001	**0.003**	0.0003	0.0001	**<0.001**
**Non-smoker**	-1.242	0.586	**0.034**			
**Sleep (hours/24hr period)**	-0.629	0.106	**<0.001**			
**Average sitting time (mins/day)**	0.002	0.001	**<0.001**			
**AUDIT score**	0.152	0.029	**<0.001**			
**DUDIT score**	0.271	0.047	**<0.001**	-0.026	0.005	**<0.001**
**BMI (kg/m²)**	0.148	0.025	**<0.001**	-0.011	0.003	**<0.001**
Breakfast			**<0.001**			**0.005**
Reference category = Less than once per week						
1-2 times per week	-1.228	0.637	0.054	0.137	0.069	0.046
3-4 times per week	-2.078	0.599	0.001	0.076	0.064	0.237
5-6 times per week	-1.780	0.588	0.002	0.002	0.063	0.978
Everyday	-3.004	0.481	<0.001	0.142	0.051	0.006
**Gender**			**<0.001**			**<0.001**
Reference category = female						
Male	-2.341	0.323	<0.001	0.302	0.035	<0.001
Another gender identity	2.663	1.912	0.164	0.004	0.206	0.983
**Age**	-0.119	0.020	**<0.001**	0.009	0.002	**<0.001**
**Non-ATSI**	-2.324	0.839	0.006			
**Living situation**			**<0.001**			**<0.001**
Reference category = Parents home						
Own home	-1.738	0.534	0.001	0.259	0.057	<0.001
On-campus	-1.167	0.549	0.034	0.079	0.058	0.176
Renting	-0.117	0.345	0.735	0.067	0.037	0.070
Boarding/Homestay	1.723	1.009	0.088	-0.057	0.109	0.599
Irregular	2.758	1.450	0.057	-0.189	0.156	0.227
**Not receiving financial support**	-0.812	0.296	0.006	0.090	0.032	0.005
**Type of degree**			**0.004**			
Reference category = Undergraduate						
Postgraduate	-1.239	0.376	0.001			
Other [b]	-0.736	0.896	0.411			
**Faculty of study**			**<0.001**			**<0.001**
Reference category = Health and Medicine						

Business and Law	0.424	0.454	0.351	-0.064	0.049	0.190
Education and Arts	1.806	0.373	<0.001	-0.207	0.040	<0.001
Engineering	1.381	0.496	0.005	-0.160	0.053	0.003
Science	1.209	0.450	0.007	-0.139	0.048	0.004
English Language and Foundation Studies	2.247	1.052	0.033	-0.214	0.069	0.002
**Physical activity (mins/week)**	-0.001	0.001	**0.002**	0.0003	0.0001	**<0.001**
**Sleep (hours/24hr period)**	-0.557	0.107	**<0.001**			
**Average sitting time (mins/day)**	0.002	0.001	**<0.001**			
**AUDIT score**	0.161	0.029	**<0.001**			
**DUDIT score**	0.303	0.045	**<0.001**	-0.027	0.005	**<0.001**
**BMI (kg/m²)**	0.148	0.025	**<0.001**	-0.012	0.003	**<0.001**

[a] β-Coefficient indicates the increase in psychological distress or resilience score per unit increase in the dietary intake variable. Higher psychological distress score indicates higher psychological distress, higher resilience score indicates higher resilience. [b] Includes students enrolled in enabling (i.e. transition to university) courses and English language courses for international students. Significant p-values in **bold**.
